# Impact of bone marrow mesenchymal stem cell immunomodulation on the osteogenic effects of laponite

**DOI:** 10.1186/s13287-018-0818-0

**Published:** 2018-04-11

**Authors:** Tao Li, Zhong Long Liu, Ming Xiao, Ze Zheng Yang, Ming Zheng Peng, Cui Di Li, Xiao Jun Zhou, Jin Wu Wang

**Affiliations:** 1 Shanghai Key Laboratory of Orthopaedic Implant, Department of Orthopaedic Surgery, Shanghai Ninth People’s Hospital, Shanghai Jiao Tong University School of Medicine, Room 701, No. 3 Building, 639 Zhizaoju Road, Shanghai, 200011 People’s Republic of China; 2 Department of Oral Maxillofacial & Head and Neck Oncology, Shanghai Ninth People’s Hospital, Shanghai Jiao Tong University School of Medicine, Shanghai, People’s Republic of China; 3 School of Biomedical Engineering, Shanghai Jiao Tong University, Shanghai, People’s Republic of China

**Keywords:** Bone marrow mesenchymal stem cell, Macrophage, Laponite, Immunomodulation, Osteogenesis

## Abstract

**Background:**

With the development of osteoimmunology and bone tissue engineering (BTE), it has been recognized that the immunomodulatory properties of bone biomaterials have considerable impact in determining their fate after implantation. In this regard, the polarization of macrophages secondary to biomaterials is postulated to play a crucial role in modulating their osteogenesis; thus, strategies that may facilitate this process engender increasing levels of attention. Whereas a variety of reports highlight the immunomodulation of bone marrow mesenchymal stem cells (BMSCs) in cell therapy or their osteogenesis in BTE, few have focused on the effect of BMSCs in promoting osteogenesis in BTE through regulating the phenotype of macrophages. Accordingly, there is an urgent need to clarify the immunomodulatory properties of agents such as laponite (Lap), which is comprised of bioactive silicate nanoplatelets with excellent osteogenesis-inducing potential, to enhance their use in BTE.

**Methods:**

In the present study, we analyzed the osteoimmunomodulatory properties of Lap alone, as well as following the introduction of BMSCs into Lap, to determine whether BMSCs could modulate its immunomodulatory properties and promote osteogenesis.

**Results:**

It was found that the BMSCs reversed the polarization of murine-derived macrophage RAW 264.7 cells from M1 as induced by pure Lap to M2 and promoted osteogenesis. In vivo study confirmed that BMSCs combined with Lap initiated a less severe immune response and had an improved effect on bone regeneration compared with Lap alone, which corresponded with the in vitro evaluation.

**Conclusion:**

These results suggest that BMSCs could ameliorate the inflammation induced by Lap and enhance its bone formation. The immunomodulatory characteristics of BMSCs suggest that these might be tailored as a new strategy to promote the osteogenic capacity of biomaterials.

## Background

Bone tissue engineering (BTE), comprising biomaterials, cell sources, and biologically active molecules, aims to reconstruct injured, lost, or destructed bone. During the past several decades, various highly innovative strategies in tissue engineering have been developed that provide promising therapies. However, before these strategies can be used in clinical applications, a comprehensive analysis of host response following delivery still needs to be performed [1].

As exogenous bodies, implants tend to be rejected or isolated by the human body, mainly through the foreign body reaction accompanied by strong inflammatory responses [2]. Macrophages, which are vital regulators of the foreign body reaction against tissue-engineered implants, has been used as model cells for the evaluation of the immune response. Recently, the term “osteoimmunomodulatory” was flagged by Chen et al. to characterize the immunomodulatory property of bone biomaterials, with a “multiple cell types” approach with the inclusion of macrophages being proposed to make up the shortcomings of traditional “one cell type” approaches when assessing the ability of materials to stimulate osteogenesis in vitro [3]. In response to different local microenvironments, macrophages can be phenotypically polarized into different forms (proinflammatory (M1) or anti-inflammatory (M2)) from primary macrophages (M0) [4]. M1 macrophages, which play significant role in the initiation of inflammation and clearance of intracellular pathogens, are characterized by high amounts of proinflammatory cytokines, such as tumor necrosis factor alpha (*TNF-α*), interleukin (IL)-*1β*, *IL-6*, and either interferon-gamma (*IFN-γ*) or *IL-12* [5]. In comparison, M2 macrophages, which are vital to the resolution of inflammation and promoting tissue remodeling, are associated with high levels of the anti-inflammatory cytokine arginase 1 (*Arg1*),* IL-1ra*, and *IL-10* [6]. In addition, the phenotypes of macrophages may be switched under certain circumstances and each subtype plays an irreplaceable role in tissue regeneration [7]. Although the underlying mechanisms by which macrophages direct the process of tissue remodeling remain unclear, it has been proposed that a timely and effective phenotypic shift from the M1 towards M2 macrophage subtype constitutes a key aspect in tissue remodeling which facilitates functional outcomes instead of scar tissue formation [1].

Based on the heterogeneity and plasticity of macrophages, several strategies have been proposed to facilitate macrophage polarization since such cells are beneficial to further promoting the osteogenic capacity of biomaterials [1]. One strategy relies upon the modification of the properties of biomaterials, such as composition, scaffold surface chemistry, and structural characteristics [1, 8, 9]. For example, Zhang et al. suggested that submicrometer bioactive glasses substituted with strontium might modulate macrophage responses for improved bone regeneration [8]. Another approach through which biomaterials can be processed to modulate the polarization of macrophages is the application of biologically active molecules [1, 10]. Liu et al. pointed out that local delivery of aspirin inhibited *IFN-γ *and *TNF-α* activities, which facilitated the shift of macrophage phenotypes and promoted bone regeneration [11]. However, despite these improved results, conflicting results associated with material modification [1], high cost, and the complex process of linking cytokines to materials [12] render these strategies less attractive.

Mesenchymal stem cells (MSCs), a group of multipotent adult stem cells capable of differentiating into multiple lineages under different stimuli and culture conditions, have long been studied for their regenerative potential in tissue engineering applications [13]. Recently, studies have shown that the therapeutic effects of MSCs in cell therapy are mainly attributed to their paracrine effects in response to the local microenvironment of injured host tissue rather than from directly differentiating into new tissues [14, 15]. Among these paracrine effects, the modulation of the macrophage phenotype switch to M2 and the beneficial remodeling events following this transition play a particularly crucial role in tissue engineering and have attracted increasing amounts of attention [16–19]. For example, cellular therapy based on MSC-mediated M2 macrophage polarization has been demonstrated to be vital in promoting tissue regeneration or repair in kidney ischemia-reperfusion injury, myocardial infarction, and acute spinal injury [20–22]. Furthermore, it has been shown that MSC-seeded constructs can also ameliorate the material-induced inflammation and promote tissue reconstruction via the M2 phenotype switch as well. This phenomenon has been shown in the field of cartilage or Achilles tendon segmental defects [4, 23]. However, few studies have focused on the role of MSCs in modulating the osteoimmunology of bone biomaterials. Based on the immunomodulatory properties of MSCs, it is a logical extension that MSCs may also represent a valuable strategy to regulate the osteoimmunomodulation of biomaterials to further promote osteogenesis.

Laponite (Lap; Na^+0.7^[(Si_8_Mg_5.5_:Li_0.3_)O_20_(OH)_4_]^−0.7^), comprised of bioactive silicate nanoplatelets, has recently received considerable attention because of its excellent osteogenesis-inducing potential [24, 25]. It has been reported that Lap could induce the osteogenic differentiation of human bone marrow mesenchymal stem cells (hBMSCs) in the absence of any osteoinductive factors [24]. Although these discoveries have fostered the application of new bioactive nanomaterials for bone tissue engineering, the effect of Lap on the immune system is unclear, especially following implantation in vivo. In addition, concomitant with an increase in the concentration of Lap, a robust production of reactive oxygen species (ROS) was observed, which has been closely associated with the development of inflammation [24]. Thus, there is an urgent need to investigate the immune reaction induced by Lap and to test the consistency of its osteogenic capacity effected by the traditional “one cell type” evaluation system with that of the new “multiple cell type” evaluation system involving macrophages. Furthermore, based on the osteoimmunomodulatory property of Lap, identifying a strategy that can deliberately modulate Lap-induced inflammation is of major concern for facilitating the regeneration of tissue-engineered bone.

In this study, we first evaluated the osteoimmunomodulatory property of Lap on murine-derived macrophage RAW264.7 cells, and then we cocultured rat BMSCs (rBMSCs) with RAW264.7 cells in the presence of Lap and hypothesized that the rBMSCs could modulate the phenotype switch of macrophages stimulated by Lap to further enhance the osteogenesis of Lap in vitro and in vivo. To verify this assumption, systematic studies were carried out by in vitro biological assessment and in vivo histological evaluation to obtain a better understanding of the interaction between Lap, BMSCs, and the immune response, as well as the effects of the immune microenvironment induced by BMSCs on the osteogenesis of biomaterials.

## Methods

### Cell culture

The murine-derived RAW 264.7 cell line (RAW264.7 cells) and rBMSCs were used in this study. RAW cell cultures were purchased from Shanghai Institutes for Biological Science, Chinese Academy of Science (Shanghai, China) and incubated in Dulbecco’s modified Eagle’s medium (DMEM; Hyclone, Logan UT, USA) supplemented with 10% fetal bovine serum (FBS; Gibco BRL, Gaithersburg, MD, USA) and 1% (*v*/v) penicillin/streptomycin (Hyclone) at 37 °C in a humidified CO_2_ incubator. At approximately 80% confluence, the growing cells were passaged by scraping and expanded through two passages before use. rBMSCs were isolated from Sprague-Dawley rats (6 weeks old; Jie Si Jie Laboratory Animal Co. Ltd., Shanghai, China) and cultured based on protocols from previous studies [26, 27]. The procedure was in compliance with the Institutional Animal Care and Use Committees (IACUC) guidelines. The obtained cells were seeded into tissue culture flasks containing DMEM supplemented with 10% FBS and 1% penicillin/streptomycin and incubated in a humidified atmosphere of 5% CO_2_ at 37 °C. The culture medium was changed the next day. The primary mesenchymal cells were expanded when the confluence reached 80% and only early passages (p3–5) of cells were used in this study.

### Preparation of Lap

Lap (Rockwood Chemical Co., Brawley, CA, USA) with low heavy metal content was kindly gifted by Tong Ji University. The transmission electron microscopy (TEM) images of Lap were obtained using a JEM-2100F (Jeol Ltd., Tokyo, Japan) transmission electron microscope operating at 200 kV. The sample was prepared by dispersing Lap in water/ethanol solution and then placing a drop on the TEM grid and allowing it to dry in vacuum. Lap dissolved in normal medium (DMEM supplemented with 10% FBS and 1% penicillin/streptomycin) at different concentrations (0, 12.5, 25, 50, 75, 100, 200, 400, 800, and 1600 μg/mL) was prepared for in vitro experiments.

### Lap cytotoxicity against rBMSCs

The cytotoxicity of different concentrations of Lap (range 0–1600 *μ*g /mL) against rBMSCs was evaluated using the Cell Counting Kit-8 (CCK-8) assay. rBMSCs were plated in a 48-well plate at a density of 2 × 10^3^ cells per well and cultured for 12 h. Then the medium was removed and subsequently replaced with Lap solution. At preselected time points (days 1, 3, and 5), 20 μL of CCK-8 stock solution was added into the medium in each well. After 2 h culture, 100 *μ*L of the reaction solution was extracted and the absorbance at the wavelength of 450 nm was measured using a microplate reader (Multiskan GO, Thermo, USA).

### In vitro effect of Lap on osteogenic differentiation of rBMSCs

#### Osteogenesis-related gene expression in rBMSCs

The expression of the osteogenesis-related genes (*ALP*, *RUNX2*, *OCN*, *OPN*, *BSP*, and *COL-1*) was detected in rBMSCs. Cells were seeded at a density of 1.5 × 10^5^ cells per well in six-well plates with culture medium. After reaching 80% confluence, the medium was refreshed with culture medium containing different concentrations of Lap (12.5, 50, 100 μg/mL). Cells cultured in the complete culture medium alone (Lap 0 *μ*g/mL) were set as the negative control. After culture for 3 days, the stimulated rBMSCs were washed twice with phosphate-buffered saline (PBS) and then the total RNA was extracted using TRIzol reagent (Invitrogen, Carlsbad, USA). Quantitative reverse transcription-polymerase chain reaction (RT-qPCR) was used to analyze the mRNA expression of target genes. Complementary DNA was synthesized from 500 ng total RNA using the DNAmo™ cDNA Synthesis Kit (Finnzymes, Thermo Scientific). RT-qPCR primers used in this study are listed in Table 1. SYBR Premix Ex Tag kit (TaKaRa Biotechnology, Shaiga, Japan) was used for qPCR with an ABI 7500 Sequencing Detection System (Applied Biosystems, Foster City, CA, USA) applied to assay the expression of the target genes. The mean cycle threshold (Ct) value of each target gene was obtained by normalizing with that of a housekeeping gene (*GAPDH*). The 2^–ΔΔCt^ method was used to determine the mRNA expression levels of each group.

**Table 1:** Osteogenesis and oncostatin M (OSM) pathway-related gene primer pairs in rat bone marrow mesenchymal stem cells used in quantitative reverse transcription-polymerase chain reaction

Gene	Primer sequences
*ALP*	Forward: 5′-TCACTTCCGCCCGGAACCCT-3
	Reverse: 5′-TGTCCTGCCGGCCCAAGAGA-3′
*RUNX2*	Forward: 5′-GCGGACGAGGCAAGAGTT-3′
	Reverse: 5′-TTGGTGCTGAGTTCAGGGAG-3′
*OCN*	Forward: 5′-TGAGGACCCTCTCTCTGCTC-3′
	Reverse: 5′-GGGCTCCAAGTCCATTGTT-3′
*OPN*	Forward: 5′-ATCTGAGTCCTTCACTG-3′
	Reverse: 5′-GGGATACTGTTCATCAGAAA-3′
*BSP*	Forward: 5′-ATAGGCAACGAGTACAACAC-3′
	Reverse: 5′-GTATCCAGATGCAAAGACAG-3′
*COL-1*	Forward: 5′-TGTTCGTGGTTCTCAGGGTAG-3′
	Reverse: 5′-TTGTCGTAGCAGGGTTCTTTC-3′
*OSMR*	Forward: 5′-TGCGGACAGAGAATGGAAGA-3′
	Reverse: 5′-GACTCCGTTGGATTGGCTTC -3′
*IL6st*	Forward: 5′-TCCACATGGCATACACA −3′
	Reverse: 5′-GCTAAGCACACAGGCACGAC -3′
*STAT3*	Forward: 5′-AGTCACACGCCACTCTGGTG −3′
	Reverse: 5′-GCTAAGCACACAGGCACGAC -3′
*GAPDH*	Forward: 5′-TGCACCACCAACTGCTTAGC-3′
	Reverse: 5′-GGCATGGACTGTGGTCATGAG-3′

#### Alkaline phosphatase (ALP) activity of rBMSCs

ALP staining and quantitative analysis was performed as previously described [28, 29]. In brief, rBMSCs were plated onto 24-well plates at a density of 5.0 × 10^3^ cells per well. At approximately 80% confluence, the culture medium was replenished with Lap solutions (0, 12.5, 50, and 100 μg/mL) containing osteogenic supplements (10 nM dexamethasone, 100 mM l-ascorbic acid 2-phosphate, 2 mM β-glycerophosphate; Sigma-Aldrich, NSW, Australia) and refreshed every 3 days. After 7 days of stimulation, the cells were fixed with 4% paraformaldehyde (PFA), and ALP staining was performed using an ALP kit (Beyotime, Shanghai, China) according to the manufacturer’s instructions. For semiquantitative analysis of ALP activity, the cells were lysed using RIPA lysis buffer (Beyotime). For ALP quantification, an ALP microplate test kit (Nanjing Jiancheng Bioengineering Institute, Nanjing, China) was used according to the manufacturer’s instruction. The quantity of ALP in the cell lysates was measured at 520 nm using a microplate reader and then this was normalized to the corresponding total protein content assessed using a BCA protein assay kit (Beyotime) following the manufacturer’s protocol.

#### Mineralization of rBMSCs

Alizarin Red S staining and semiquantitative analysis was used to highlight mineralization nodules produced by BMSCs grown for 14 days in Lap (0, 12.5, 50, and 100 μg/mL). The cells were gently rinsed with ddH_2_O three times and then were fixed in 4% PFA for 10 min at room temperature. A solution of 1% Alizarin Red S (pH 4.2, Sigma-Aldrich) was added and kept for 10 min to visualize the nodules. Afterward, the cells were washed thoroughly with PBS and images were captured using light microscopy. Quantitative analysis of Alizarin Red S staining was performed by eluting the bound stain with 10% cetylpyridinium chloride for 1 h. The absorbance of the resulting solution at 562 nm was determined using a microplate reader (Thermo3001).

### Effects of Lap on macrophage phenotype switching

#### Proliferation of RAW264.7 cells with Lap

RAW264.7 cells were plated onto a 96-well plate at a density of 2 × 10^3^ cells per well. After being cultured for 1, 3, and 5 days, the cells were subjected to CCK-8 assays to evaluate their proliferation as described above for rBMSCs.

#### Morphological changes of RAW264.7 cells stimulated by Lap

Lap was labeled with rhodamine B prior to evaluating its influence on the morphological changes of RAW264.7 cells. Briefly, 1 g of Lap was dissolved in 50 mL of 0.1% (wt/v) rhodamine B isothiocyanate (Sigma) solution. Then the mixture was washed with ddH_2_O 5–6 times to remove the excess rhodamine B. Finally, Lap was air-dried and stored at room temperature and protected from light until further use. RAW264.7 cells were plated onto a confocal plate (diameter 4 cm) at a density of 1 × 10^5^ cells/cm^2^ and allowed to attach overnight. Then the culture medium was refreshed with DMEM containing rhodamine-labeled Lap (12.5, 50, and 100 μg/mL); cells cultured in the complete culture medium alone (Lap 0 *μ*g/mL) were taken as the negative control. The cultures were maintained and protected from light at 37 °C in a humidified CO_2_ incubator for an additional 48 h. Subsequently, the culture medium was removed and cells were washed with PBS gently three times, fixed in 4% PFA, and permeabilized with 0.1% Triton X-100 in PBS for 10 min. The cellular F-actin was then stained with FITC-phalloidin (F432, Thermo Fisher Scientific, Waltham, MA, USA). Afterwards, the cell nuclei were counterstained with 4,6-diamidino-2-18-phenylindole (DAPI; Molecular Probe, Sigma-Aldrich). Finally, samples were visualized using confocal laser scanning microscopy (Leica, Tokyo, Japan).

#### Flow cytometry

The surface markers CCR-7 (M1 marker) and CD206 (M2 marker) were detected to analyze the polarization of the macrophages. After 3 days of incubation with culture medium containing Lap (0, 12.5, 50, and 100 μg/mL), RAW264.7 cells were physically detached and centrifuged. The cell pellets were resuspended with 1% *w/v* bovine serum albumin/PBS to block nonspecific proteins. Samples were then incubated with PE rat anti-mouse CD197 (CCR-7; 1:25, BD Pharmingen, Franklin Lakes, NJ, USA) and Alexa Fluor® 647 rat anti-mouse CD206 antibody (1:50, BD Pharmingen) for 30 min. Corresponding isotype controls were used as instructed by the manufacturer. The treated cells were analyzed using a BD LSRFortessa (BD Biosciences) and data analysis was performed using FlowJo software (TreeStar, Ashland, OR, USA). A positive control was included in this experiment by incubating RAW264.7 cells with lipopolysaccharide (LPS; 100 ng/ml) for 24 h which could polarize macrophages to the M1 phenotype.

#### Intracellular ROS production

ROS released from macrophages plays a significant part in the macrophage-elicited innate immune response by promoting the formation of foreign body giant cells and accelerating the degradation of foreign materials. However, at high levels it causes tissue damage and acts as an indicator of the detrimental effect of interactions between macrophages and materials [30]. The intracellular production of ROS by RAW264.7 cells was evaluated using a fluorometric intracellular ROS kit (Sigma, St. Louis, MO, USA), performed following the manufacturer’s instructions. Briefly, RAW264.7 cells were plated onto 96-well plates at a density of 1 × 10^4^ cells per well and cultured with Lap solutions (12.5, 50, and 100 μg/mL) for 24 h; cells cultured in the complete culture medium alone (Lap 0 *μ*g/mL) were taken as the negative control. The culture medium was removed and 100 μL test medium was added to each well. After 30 min of stimulation, cells were washed with PBS gently three times and fixed with 4% PFA for 10 min. Images were captured via fluorescence microscopy and the intracellular ROS level was examined using a fluorescence microplate reader with detection at λex = 640/λem = 675 nm.

#### Inflammatory cytokine and gene expression in RAW264.7 cells treated with Lap

RAW264.7 cells were seeded at a density of 2 × 10^5^ cells per well (six-well plate). The culture medium was replaced by Lap solutions (0, 12.5, 50, and 100 μg/mL) when the cell confluence reached 80%. After 3 days, the culture supernatants were collected and centrifuged at 250 g to remove the dead cells. The clarified supernatants were then extracted for enzyme-linked immunosorbent assay (ELISA) to analyze the content of proinflammatory (IL-6, TNF-α, IL-1β, IFN-γ) and anti-inflammatory (Arg-1, IL-1ra, IL-10) factors with ELISA kits (R&D Systems, Gymea, NSW, Australia) according to the manufacturer’s instructions. The supernatants were also mixed with osteogenic inducing medium or culture medium at a ratio of 1:2 and stored at −80 °C for the following conditioned-media experiments. In addition, total RNA of RAW264.7 cells was isolated from each group and RT-qPCR was conducted to evaluate the mRNA expression of the above factors as described previously. RT-qPCR primers are listed in Table 2.

**Table 2:** Inflammatory-related gene primer pairs in RAW cells used in quantitative reverse transcription-polymerase chain reaction

Gene	Primer sequences
*TNF-α*	Forward: 5′-CTGAACTTCGGGGTGATCGG-3′
	Reverse: 5′-GGCTTGTCACTCGAATTTTGAGA-3′
*IL-1β*	Forward: 5′-TGGAGAGTGTGGATCCCAAG-3′
	Reverse: 5′-GGTGCTGATGTACCAGTTGG-3′
*IFN-γ*	Forward: 5′-TGAAAATCCTGCAGAGCCAG-3′
	Reverse: 5′-TGGACCTGTGGGTTGTTGAC-3′
*IL-6*	Forward: 5′-ATAGTCCTTCCTACCCCAATTTCC-3′
	Reverse: 5′-GATGAATTGGATGGTCTTGGTCC-3′
*IL-10*	Forward: 5′-GAGAAGCATGGCCCAGAAATC-3′
	Reverse: 5′-GAGAAATCGATGACAGCGCC-3′
*IL-1ra*	Forward :5′-CTCCAGCTGGAGGAAGTTAAC-3′
	Reverse: 5′-CTGACTCAAAGCTGGTGGTG-3′
*Arg-1*	Forward: 5′-CATATCTGCCAAGGACATCG-3′
	Reverse: 5′-GGTCTCTTCCATCACTTTGC-3′
*BMP-2*	Forward: 5′-GCTCCACAAACGAGAAAAGC-3′
	Reverse: 5′-AGCAAGGGGAAAAGGACACT-3′
*OSM*	Forward: 5′-AACTCTTCCTCTCAGCTCCT-3′
	Reverse: 5′-TGTGTTCAGGTTTTGGAGGC-3′
*VEGF*	Forward: 5′-GTCCCATGAAGTGATCAAGTTC-3′
	Reverse: 5’-TCTGCATGGTGATGTTGCTCTCTG-3′
*TGF-β1*	Forward: 5′-CAGTACAGCAAGGTCCTTGC-3′
	Reverse: 5′-ACGTAGTAGACGATGGGCAG-3′
*TGF-β3*	Forward: 5′-CAACACCCTGAACCCAGAG-3′
	Reverse: 5′-CTTCACCACCATGTTGGACAG-3′
*GAPDH*	Forward: 5′-CTCCCACTCTTCCACCTTCG −3′
	Reverse: 5′-TTGCTGTAGCCGTATTCATT −3′

#### Osteogenesis of Lap/macrophage-conditioned medium

rBMSCs were seeded in plates at a density of 5 × 10^3^ cells/cm^2^. At approximately 80% confluence, the medium was replenished by four groups of macrophage-conditioned media (MΦCM) mixed with osteogenic supplements at a ratio of 1:2 as described previously. Then, osteogenesis-related genes of rBMSCs, ALP staining and activity, and Alizarin Red S staining in response to macrophage-conditioned Lap were evaluated as described previously.

### Effects of RAW/rBMSC coculture on macrophage phenotype switching

#### Effects of Lap on cell morphology, ROS production, and flow cytometry of RAW264.7 cells upon coculture with rBMSCs

To assess BMSC modulation of the osteoimmunomodulatory properties of Lap, rBMSCs were indirectly cocultured with RAW264.7 cells. We examined three groups: group one, RAW264.7 cells were indirectly cocultured with rBMSCs without Lap (rBMSC/RAW); group two, RAW264.7 cells were cultured with Lap (100 μg/mL) without rBMSCs (Lap/RAW); and group three, RAW264.7 cells were cultured with Lap (100 *μ*g/mL) with rBMSC indirect coculturing (Lap/rBMSC/RAW). For the coculture systems, RAW264.7 cells at a density of 2.0 × 10^5^ were plated in the lower chambers of transwell plates (0.4 mm pore size; Corning Costar, Cambridge, MA, USA). rBMSCs were added into the upper chambers of the transwell plates at a 1:10 ratio of RAW264.7 cells. For RAW cell morphological change analysis, Lap (100 *μ*g/mL) was first labeled with rhodamine B as described above. At 48 h after incubation, the morphological change of RAW264.7 cells was examined. After indirect coculture for 3 days, the production of ROS and flow cytometry characteristics were also assessed as described above.

#### Inflammation-related osteogenic gene and cytokine expression in the coculture system

RAW264.7 cells were indirectly cocultured with rBMSCs as described above. After 3 days, the supernatants from the three groups were collected and centrifuged at 250 g. ELISA analyses for IL-6, TNF-α, IL-1β, IFN-γ, Arg-1, IL-1ra, IL-10, bone morphogenetic protein (BMP)-2, oncostatin M (OSM), vascular endothelial growth factor (VEGF), transforming growth factor (TGF)-β1, and TGF-β3 from the conditioned media were then performed following the manufacturer’s instructions as described above. For inflammation-related gene expression analysis and metabolism analysis, total RNA from RAW264.7 cells and rBMSCs was isolated. The proinflammatory (*IL-6, TNF-α, IL-1β, IFN-γ*), anti-inflammatory (*Arg-1, IL-1ra, IL-10*), osteo-related (*BMP-2, OSM*), and fibrous-related (*VEGF, TGF-β1*, and *TGF-β3*) gene expression assessment in RAW264.7 cells was conducted as described above. Gene expression of the indicators of the OSM signaling pathway (Osmr, Il6st, Stat3) in rBMSCs were also assessed. The subsequent BMSC cocultured macrophage-conditioned material experiments were performed by using a mixture of the above supernatants and osteogenic-inducing medium at a ratio of 1:2.

#### Effect of Lap-stimulated macrophage-conditioned medium in promoting the osteogenesis of BMSCs

The expression of osteogenesis-related genes (*ALP, RUNX2, OCN, OPN, BSP, COL-1*), ALP activity, Alizarin Red S staining, and semiquantitative analysis of rBMSCs treated with different MΦCM were evaluated as described above.

### In vivo studies

#### Surgical procedure and treatment

Surgical procedures were performed on Sprague-Dawley rats (8 weeks old, female, weight 180–200 g) in accordance with a previously reported method [3]. All of the animal experimental protocols were approved by the Institutional Animal Care Committee of Shanghai Ninth People’s Hospital affiliated with Shanghai Jiao Tong University.

Absorbable gelatin sponge pillars (Jinling, Nanjing, China; 3 mm wide × 3 mm deep) generated using an electric trephine drill (Saeshin, Taegu, South Korea) were immersed in Lap solution (100 μg/mL) on a shaking table overnight. After anesthesia with chloral hydrate (350 mg/kg), a 3-mm diameter × 3-mm deep bone defect was made on the lateral condyle of the femur with the trephine drill. The Lap-containing pillars were then deposited into the defects. For the Lap+rBMSC group, 2 × 10^5^ rBMSCs were injected into the defect after the Lap sponge was deposited into the defect. The wounds were sutured and prophylactic antibiotic was administered to avoid infection. All rats were sacrificed after 4 weeks. The femoral condyles were harvested and fixed in 10% formaldehyde.

#### Histological analysis

Femoral bone samples were decalcified by immersing samples in decalcifying fluid for 4 weeks. Then, 5-μm thick sections were cut from the paraffin-embedded tissues to conduct the histological evaluation. Hematoxylin and eosin (H&E) staining was performed for these sections and images were captured under a light microscope (TE2000U, Nikon, Tokyo, Japan).

#### Immunohistochemistry

Immunohistochemistry was conducted on deparaffinized and rehydrated slides with primary antibodies against the M1 markers CCR-7 (1:500, BD Pharmingen) and CD11c (1:400, BD Pharmingen) and the M2 marker CD163 (1:500, BD Pharmingen). The stained slides were imaged using a light microscope (Nikon).

### Statistical analysis

Quantitative results are presented in the form of mean ± standard deviation (SD) with statistical significance set at *P < 0.05, **P < 0.01, and ***P < 0.001. Statistical analysis was performed using one-way analysis of variance (ANOVA) followed by LSD *post-hoc* test to compare selected data pairs.

## Results

### Effect of Lap on rBMSCs

#### Potential cytotoxic effects of Lap on rBMSCs

**Fig. 1:**
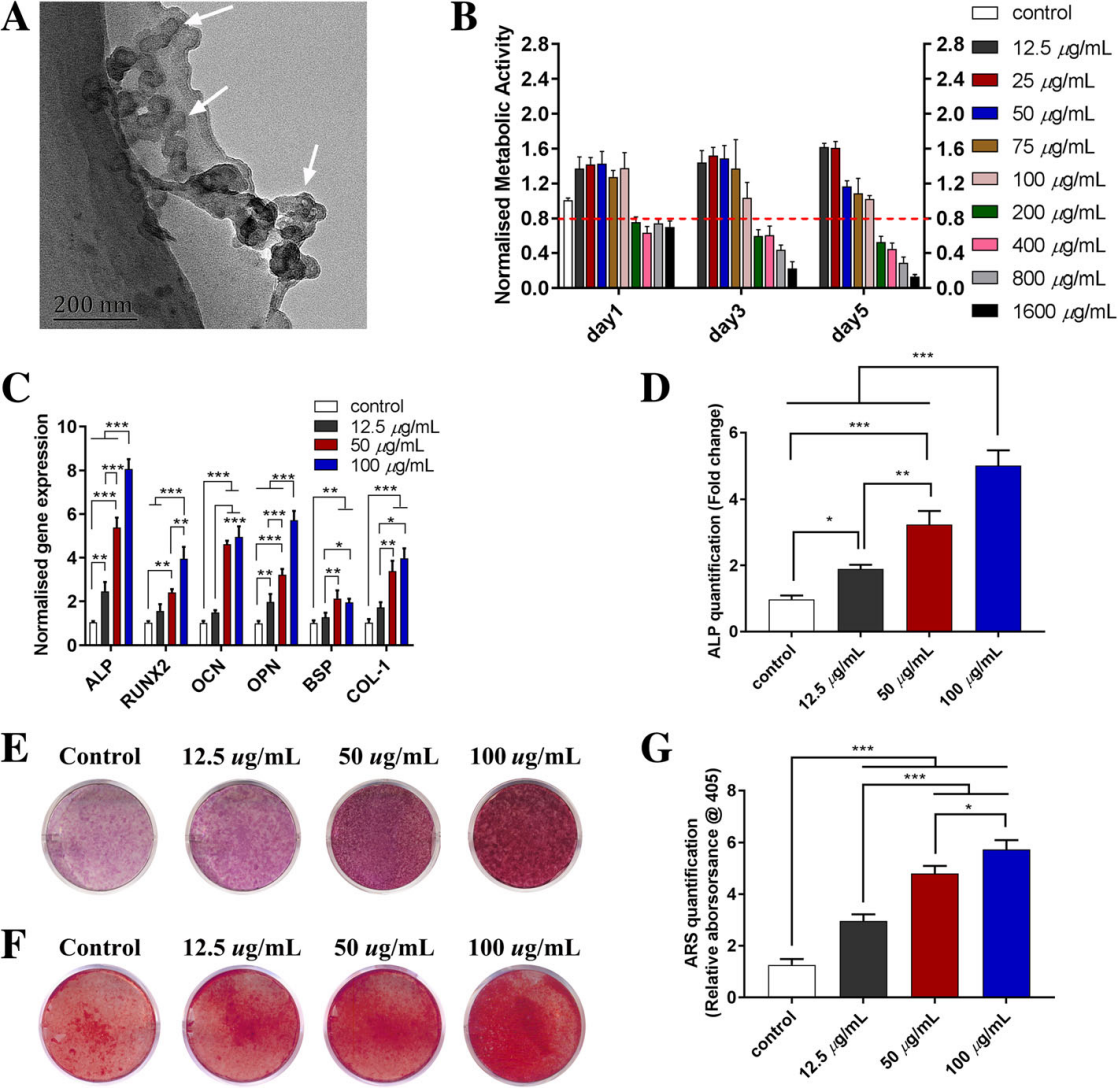
TEM image, cytotoxicity, and osteogenesis of Lap on rBMSCs. **a** TEM image of Lap (Lap indicated by white arrows). (**b**) The metabolic activity of rBMSCs cultured with different concentrations of Lap. Results were normalized against the control (Lap 0 μg/mL) from day 1. The threshold of appropriate laponite concentration was determined at the value of 80% in relation to the control. **c** mRNA expression of osteogenesis-related genes (*ALP, RUNX2, OCN, OPN, BSP, *and *COL-1*) of rBMSCs after culture with different concentrations of Lap for 3 days. Alkaline phosphatase (ALP) staining and ALP activity at day 7 (**d** and **e**), and Alizarin Red S (ARS) staining and quantitative analysis at day 14 (**f** and **g**). The experiment was repeated three times with similar results. *P < 0.05, **P < 0.01, ***P < 0.001

It has been reported that Lap presents with a disk shape, 20–30 nm in dimeter and approximately 1 nm in thickness [24]. The TEM image in Fig. 1a shows that the obtained Lap had a disc-shaped morphology with a diameter ranging from 20 to 50 nm. The metabolic activity of rBMSCs with the addition of Lap was evaluated using a CCK-8 test over a period of 5 days. The addition of Lap from 12.5 μg/mL up to 100 *μ*g/mL to the cells did not cause significant changes in their metabolic activity; however, when the concentrations of Lap was more than 100 *μ*g/mL (200–1600 *μ*g/mL), an abrupt decrease (more than 20%) in the metabolic activity of cells was observed (Fig. 1b). Previous reports indicated that Lap concentrations < 100 μg/mL did not result in significant cytotoxicity on hBMSCs or the SSEA-4 subpopulation of human adipose-derived stem cells [25, 26], which coincides with our study. Since the concentrations of Lap higher than 100 *μ*g/mL led to > 20% reduction in cell metabolic activity, the concentrations 0, 12.5, 50, and 100 *μ*g/mL were adopted for our subsequent experiments on osteogenic differentiation of rBMSCs.

#### Effects of Lap on osteogenic differentiation of rBMSCs in the “one cell type” approach

The mRNA expression of the osteogenesis-related genes (*ALP, RUNX2, OCN, OPN, BSP*, and * COL-1*) was significantly upregulated in rBMSCs in proportion to the concentration of Lap added, especially at 100 μg/mL (*P* < 0.05) (Fig. 1c). The ALP staining and ALP activity also obviously increased with the addition of Lap (Fig. 1d, e). Alizarin Red S staining (Fig. 1f) demonstrated mineralized nodules in all the groups with the addition of osteogenic supplements; however, more significant mineralization nodules were displayed in rBMSCs incubated with Lap than following pure osteogenic supplements, and were the most distinct in the 100 μg/mL group. The quantitative analysis (Fig. 1g) reconfirmed the enhancement of osteogenesis along with the concentration of added Lap from 0 to 100 *μ*g/mL.

### Effects of Lap on the macrophage phenotype switch

#### Proliferation of RAW264.7 cells with Lap

**Fig. 2:**
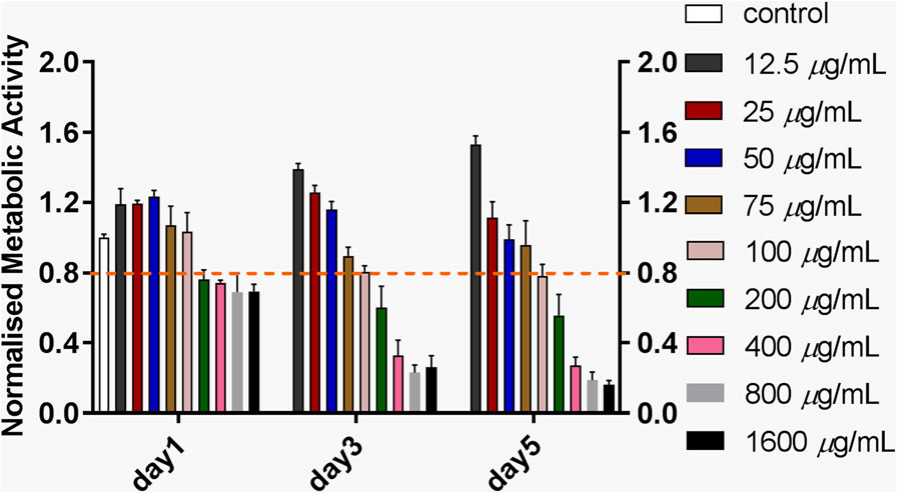
The metabolic activity of RAW264.7 cells in the presence of different concentrations of Lap. Results were normalized against the control (without any Lap) on day 1. The metabolic activity threshold for choosing the appropriate Lap concentrations was set to 80% in relation to the control. The experiment was repeated three times with similar results

The CCK-8 assays showed that with the addition of Lap from 12.5 up to 100 *μ*g/mL the metabolic activity of RAW264.7 cells was inhibited. This trend was more distinct as the culture time prolonged from day 1 to day 5. Furthermore, at higher concentrations (ranging from 200 to 1600 μg/mL) an abrupt decrease in the metabolic activity of cells (more than 20%) was observed (Fig. 2). The range of noncytotoxic concentration for RAW264.7 cells was coincident with that of rBMSCs. Thus, Lap concentrations less than 200 μg/mL (0, 12.5, 50, and 100 *μ*g/mL) were adopted for subsequent experiments.

#### Flow cytometry, morphologic change, and ROS production of RAW264.7 cells with Lap

**Fig. 3:**
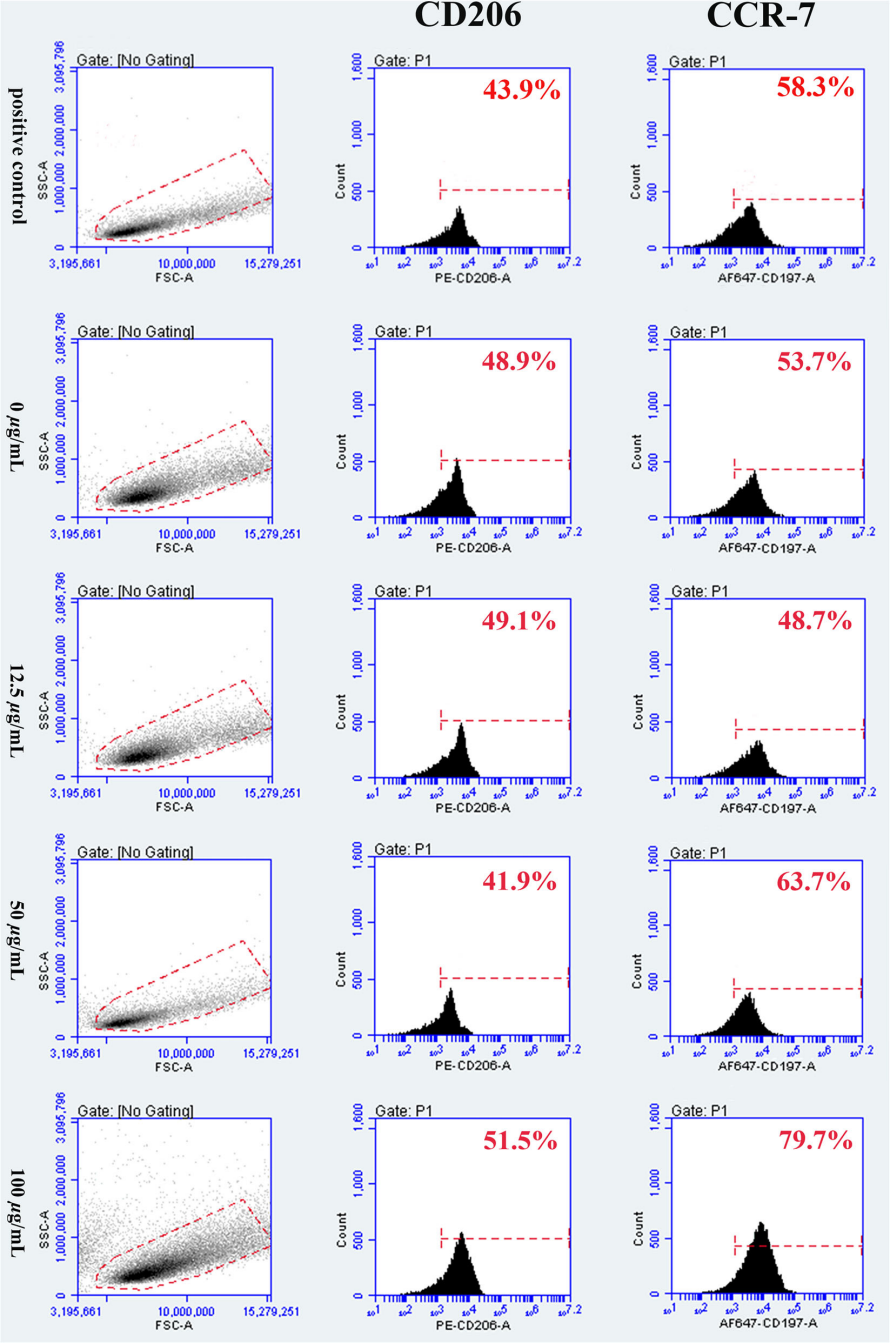
Flow cytometry results of RAW264.7 cells in M1-positive control (LPS, 100 *n*g/mL) and incubated with different concentrations of Lap (0, 12.5, 50, and 100 *μ*g/mL). The experiment was repeated three times with similar results

**Fig. 4:**
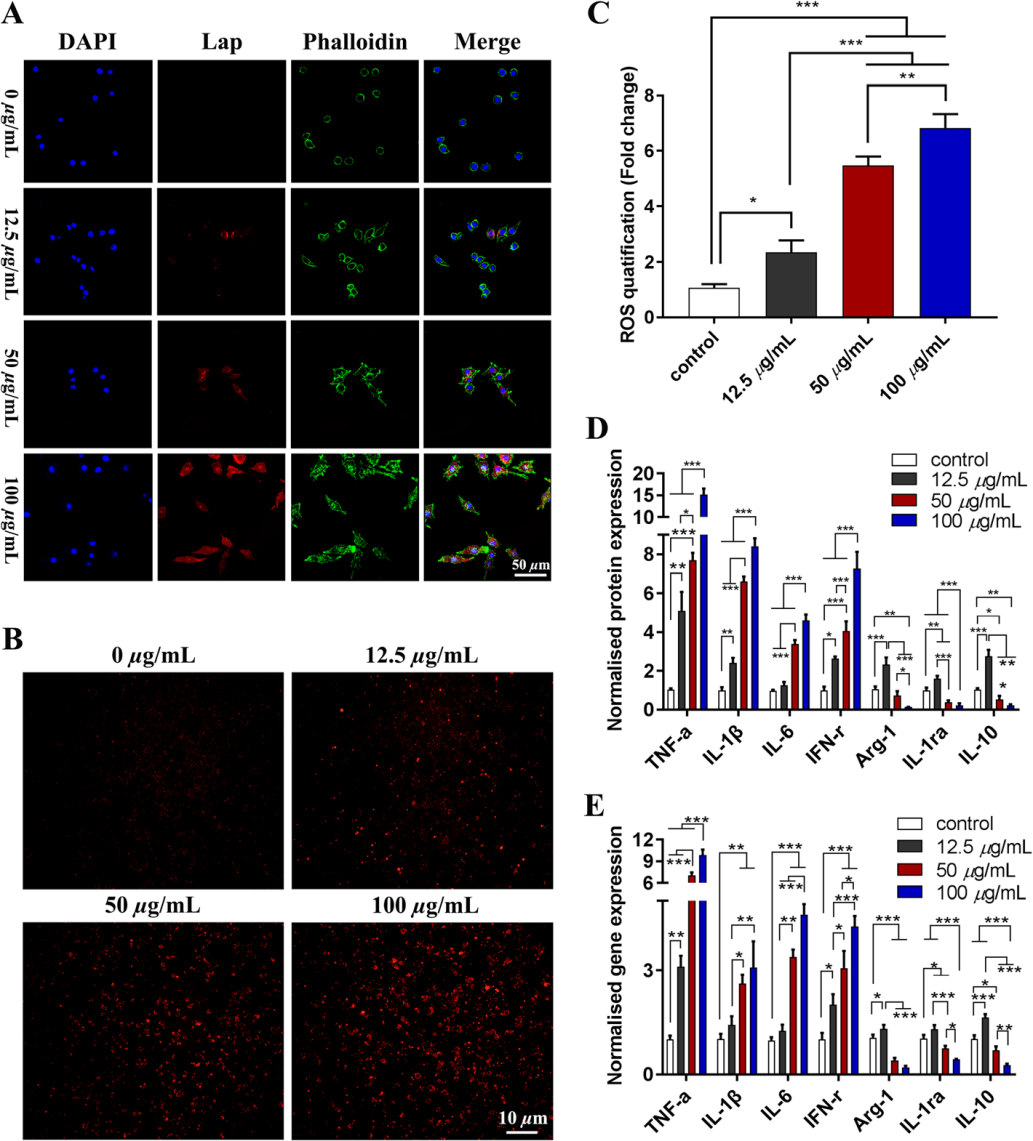
The morphological change (**a**), reactive oxygen species (ROS) staining (**b**), and quantification (**c**) of RAW264.7 cells in the control group (Lap 0 *μ*g/mL) and Lap (12.5, 50, 100 *μ*g/mL) groups. ELISA assay (**d**), and mRNA expression (**e**) of the proinflammatory (tumor necrosis factor alpha (TNF-α), interferon-gamma (IFN-γ), interleukin (IL)-6, IL-1β) and anti- inflammatory (IL-1α, IL-10, arginase 1 (Arg-1)) cytokines from RAW264.7 cells in the control and Lap groups. The experiment was repeated three times with similar results. *P < 0.05, **P < 0.01, ***P < 0.001

After being treated with different concentrations of Lap, studies of macrophage surface markers as detected by flow cytometry showed that more RAW264.7 cells expressed the M1 marker CCR-7 following stimulation by higher concentrations of Lap in comparison with cells stimulated with the control medium (Lap, 0 μg/mL). For the expression of CD206, we found that there was no significant increase after stimulation by increasing concentrations of Lap (Fig. 3). Although the expression of CD206 was downregulated in the 50 μg/ml Lap as compared with that of the control group, the cell phenotype was not changed. Collectively, the results of flow cytometry analysis reflected that Lap could polarize macrophages to the M1 phenotype at high concentration (50 μg/mL and 100 *μ*g/mL). This finding was further verified by the morphological change in RAW264.7 cells, with more synaptic structures being observed with increasing concentrations of Lap (Fig. 4a). Furthermore, the results of ROS staining and quantitative analysis also showed a similar trend (Fig. 4b, c).

#### Inflammatory gene expression in RAW264.7 cells treated with Lap

Compared with the control group (Lap 0 μg/mL), the ELISA results of the anti-inflammatory factors (IL-1ra, IL-10, and Arg-1) were significantly downregulated in RAW264.7 cells as the concentration of Lap increased from 12.5 μg/mL to 100 μg/mL (*P* < 0.05) (Fig. 4d), whereas the expression of the proinflammatory factors (TNF-α, IL-1β, IL-6, and IFN-γ) were significantly upregulated (*P* < 0.05), especially TNF-α which showed 10-times higher expression upon 100 μg/mL Lap treatment than the negative control group. mRNA analysis showed similar trends (Fig. 4e). RAW264.7 cells stimulated by Lap expressed a lower level of *IL-1ra, IL-10*, and * Arg1 *(*P* < 0.05). In contrast, mRNA expression of the proinflammatory cytokines* TNF-α, IL-1β, IL-6,* and *IFN-γ* were significantly enhanced, most significantly in the 100 μg/mL group.

#### Effects of Lap-RAW cell-conditioned medium on the osteogenic differentiation of rBMSCs

**Fig. 5:**
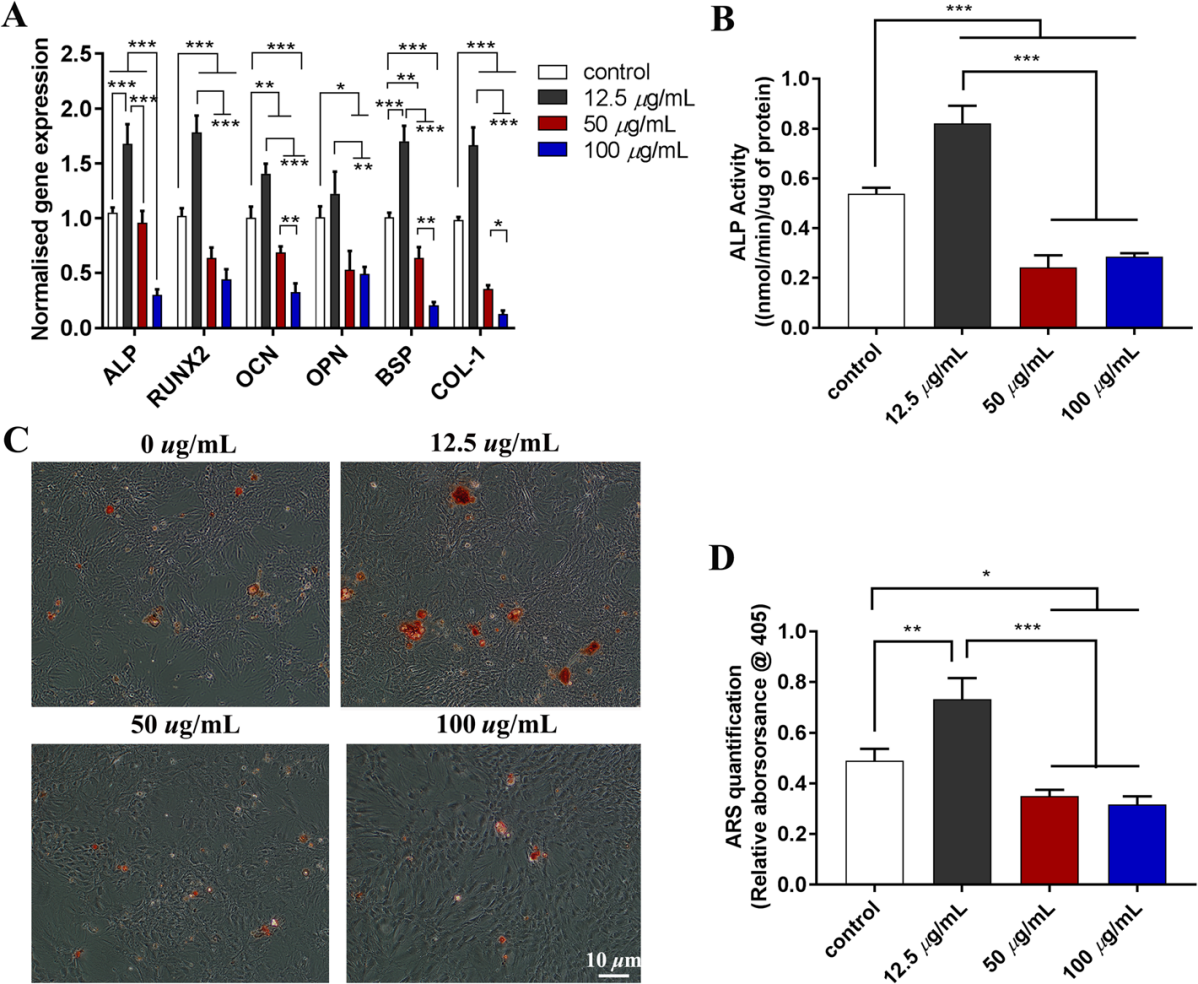
Effect of different Lap-RAW cell-conditioned medium on osteogenic differentiation of rBMSCs. **a** mRNA expression of osteogenesis-related genes (*ALP, RUNX2, OCN, OPN, BSP,* and *COL-1*) at day 3. **b** Alkaline phosphatase (ALP) activity at day 7. Alizarin Red S (ARS) staining (**c**) and quantitative results (**d**) at day 14. The experiment was repeated three times with similar results. *P < 0.05, **P < 0.01, ***P < 0.001

As shown in Fig. 5a, compared with the control group the mRNA expression of the mineralization-related genes *ALP, RUNX2, OCN*, and *OPN*, as well as *BSP* and *COL-1*, was significantly upregulated with the MΦCM in the 12.5 μg/mL Lap group. However, as the added Lap concentration increased (to 50 and 100 μg/mL) the expression of these genes was significantly decreased, even to levels below the control group. The ALP activity (Fig. 5b) or Alizarin Red S staining (Fig. 5c) and corresponding quantitative analysis (Fig. 5d) were detected in RAW264.7 cells in response to MΦCM after 7- or 14-day stimulation, respectively. Compared with the control group, the ALP activity in the 12.5 μg/mL Lap group was higher than that in the control group. Conversely, the ALP activity in the 50 and 100 μg/mL Lap groups was significantly decreased compared with the control group. Alizarin Red S staining showed the most pronounced mineralized nodules in the 12.5 μg/mL Lap group, followed by the control group, with fewer nodules in the higher concentration Lap groups (50 and 100 μg/mL). The quantitative analysis confirmed the results of Alizarin Red S staining.

### Effects of coculture on Lap-mediated polarization and osteogenesis

#### Effect of BMSCs in modulating cell morphological change, ROS production, and polarization of RAW264.7 cells stimulated by Lap

**Fig. 6:**
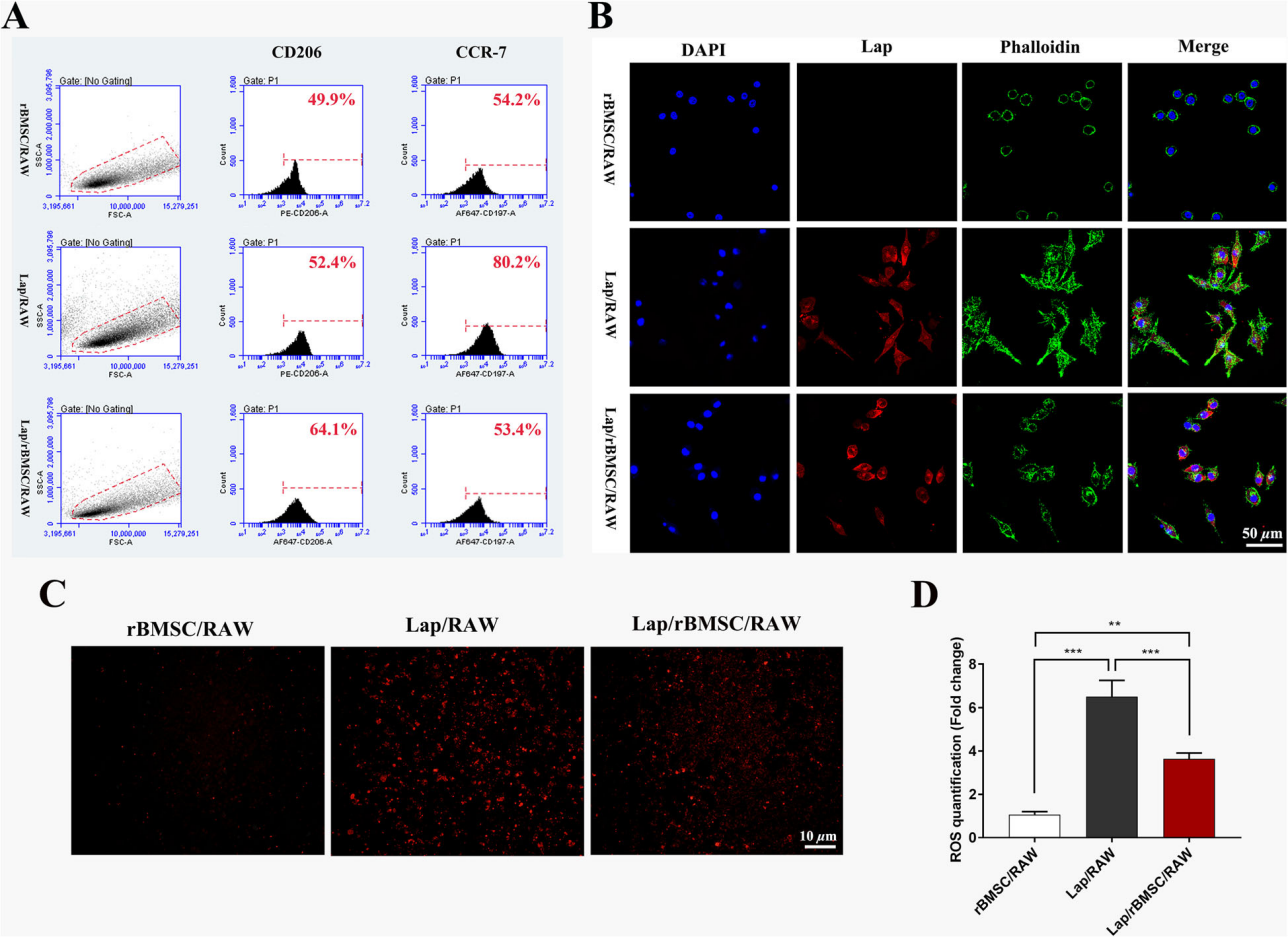
Flow cytometry results of the surface markers CD206 (M2) and CCR-7 (M1) (**a**), the morphological change in RAW264.7 cells (**b**), reactive oxygen species (ROS) staining (**c**) and quantification (**d**) in RAW264.7 cells from the rBMSC/RAW group, Lap/RAW group, and Lap/rBMSC/RAW group. The experiment was repeated three times with similar results. Lap, laponite, rBMSC rat bone marrow mesenchymal stem cell

To determine the role of BMSCs in modulating the osteoimmunomodulatory effects of Lap, indirect coculture of rBMSCs with RAW264.7 cells in the presence of Lap was applied. When cultured for 3 days, the phenotype switch of RAW264.7 cells after coculture with rBMSCs was assessed. The results showed that incubation with BMSCs markedly increased the percentage of CD206-positive macrophages. In contrast, the percentage of CCR-7-positive macrophages significantly decreased (Fig. 6a). With respect to morphological change, no significant synaptic structures were observed in RAW264.7 cells in the rBMSC/RAW group. Furthermore, compared with the Lap/RAW group, fewer synaptic structures were observed in RAW264.7 cells in the Lap/rBMSC/RAW group, although this group still exhibited some synaptic structures in comparison with the negative control group (Fig. 6b). ROS staining (Fig. 6c) and quantitative analysis (Fig. 6d) in RAW264.7 cells also demonstrated a statistically significant reduction in the Lap/rBMSC/RAW group compared with the Lap/RAW group.

#### Expression of polarization-related genes in RAW264.7 cells after coculture with BMSCs

**Fig. 7:**
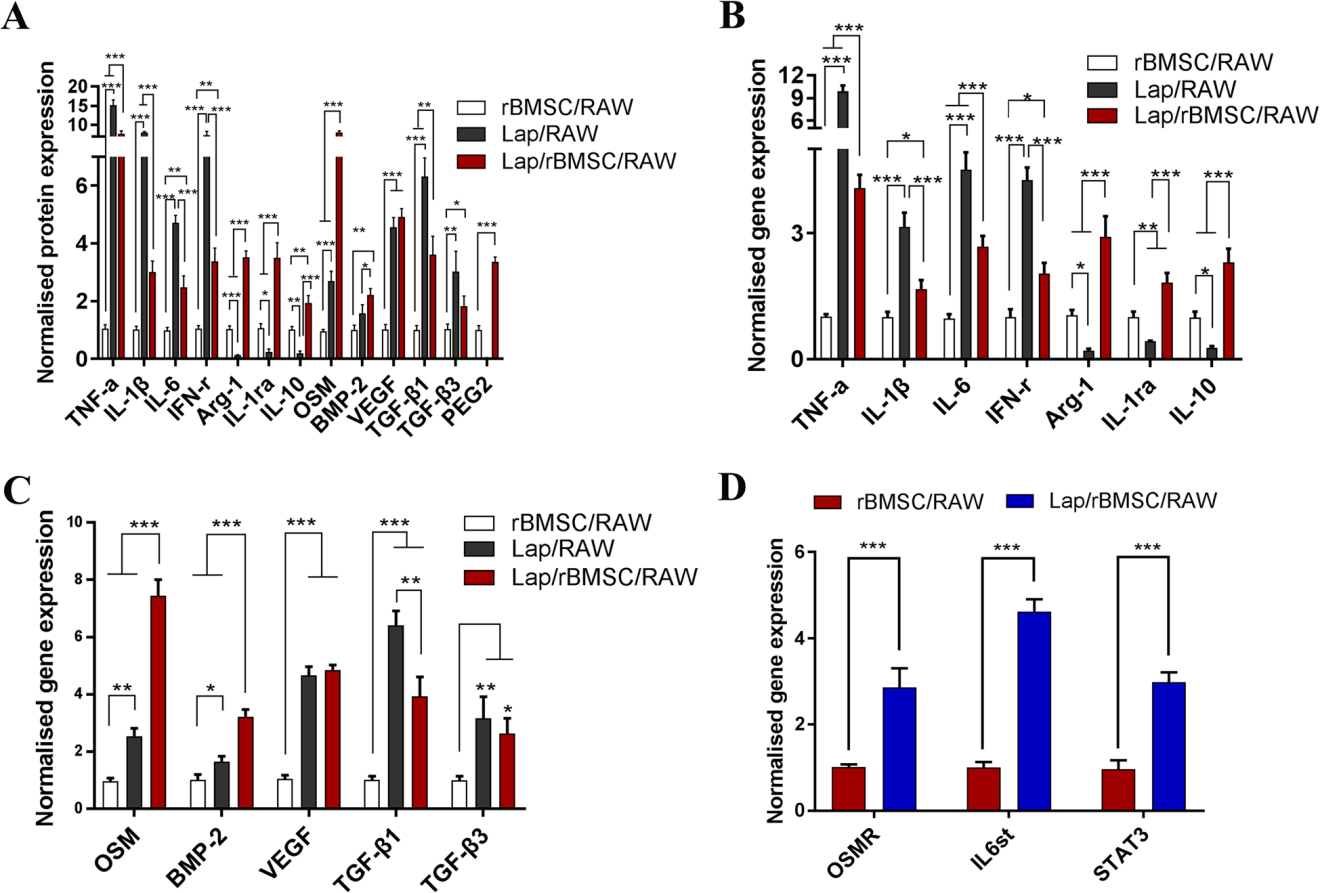
Bone marrow mesenchymal stem cells (BMSCs) reverse the polarization of macrophages stimulated by laponite (Lap). **a** ELISA assay of the proinflammatory factors (tumor necrosis factor alpha (TNF-α), interleukin (IL)-6, interferon-gamma (IFN-γ), IL-1β), anti-inflammatory factors (arginase 1 (Arg-1), IL-10, and IL-1ra), osteogenesis-related factors (oncostatin M (OSM) and bone morphogenetic factor 2 (BMP2)), fibrous gene-encoded factors (transforming growth factor (TGF)-β1, TGF-β3, vascular endothelial growth factor (VEGF)) and PEG2 released into the culture medium from the rBMSC/RAW, Lap/RAW, and the Lap/rBMSC/RAW coculture system groups. **b** Relative mRNA expression of proinflammatory (*TNF-α, IL-6, IFN-γ, IL-1β*) and anti-inflammatory genes (*Arg-1, IL-10,* and *IL-1ra*) in RAW264.7 cells. **c** Relative mRNA expression of osteogenesis-related genes (*OSM, BMP-2*) and fibrous-related genes (*TGF-β1, TGF-β3, VEGF*). **d** Gene expression of *OSMR*, *IL6st*, and *STAT3* in rat BMSCs (rBMSCs) from coculture systems of rBMSC/RAW and Lap/rBMSC/RAW. The experiment was repeated three times with similar results. *P < 0.05, **P < 0.01, ***P < 0.001

To confirm M2 macrophage switch in the coculture system, the expression of inflammation-related factors and macrophage surface markers that indicate the polarization of M1 or M2 were assessed. The results showed that the protein and mRNA expression of proinflammatory factors in RAW264.7 cells, including IL-1β, TNF-α, IL-6, and IFN-γ, was significantly downregulated in the Lap/rBMSC/RAW coculture group compared with the Lap/RAW group (Fig. 7a, b). In contrast, the expression of the anti-inflammatory cytokines IL-1ra, IL-10, and Arg-1 were significantly higher in the Lap/rBMSC/RAW group compared with the Lap/RAW group (Fig. 7a, b). With respect to the protein and mRNA expression of osteogenesis-related factors in RAW264.7 cells, BMP-2 and OSM in RAW264.7 cells from the Lap/rBMSC/RAW group were significantly upregulated when compared with the Lap/RAW group (Fig. 7a, c). PEG2, a key cytokine involved in the interaction between MSCs and macrophages, was also detected (Fig. 7a) [31]. Compared with the rBMSC/RAW group, the production of PEG2 in the Lap/rBMSC/RRAW group was significantly upregulated, whereas no detectable PEG2 was found in the Lap/RAW group. Additionally, the fibrosis-related genes *VEGF, TGF-β1*, and *TGF-β3* in the RAW264.7 cells were analyzed (Fig. 7c). The expression of *VEGF* showed no statistical difference; however, the expressions of *TGF-β1 *and *TGF-β3* were significantly downregulated. Furthermore, quantification of the expression of the OSM pathway-related genes* OSMR, IL6st,* and *STAT3* in rBMSCs in the group of rBMSC/RAW was statistically higher than that in the group of Lap/rBMSC/RAW (Fig. 7d) which indicates that the activation of the OSM pathway is likely involved in the enhanced osteogenesis by rBMSCs.

#### Osteogenesis ability of Lap/BMSC/RAW cell-conditioned medium

**Fig. 8:**
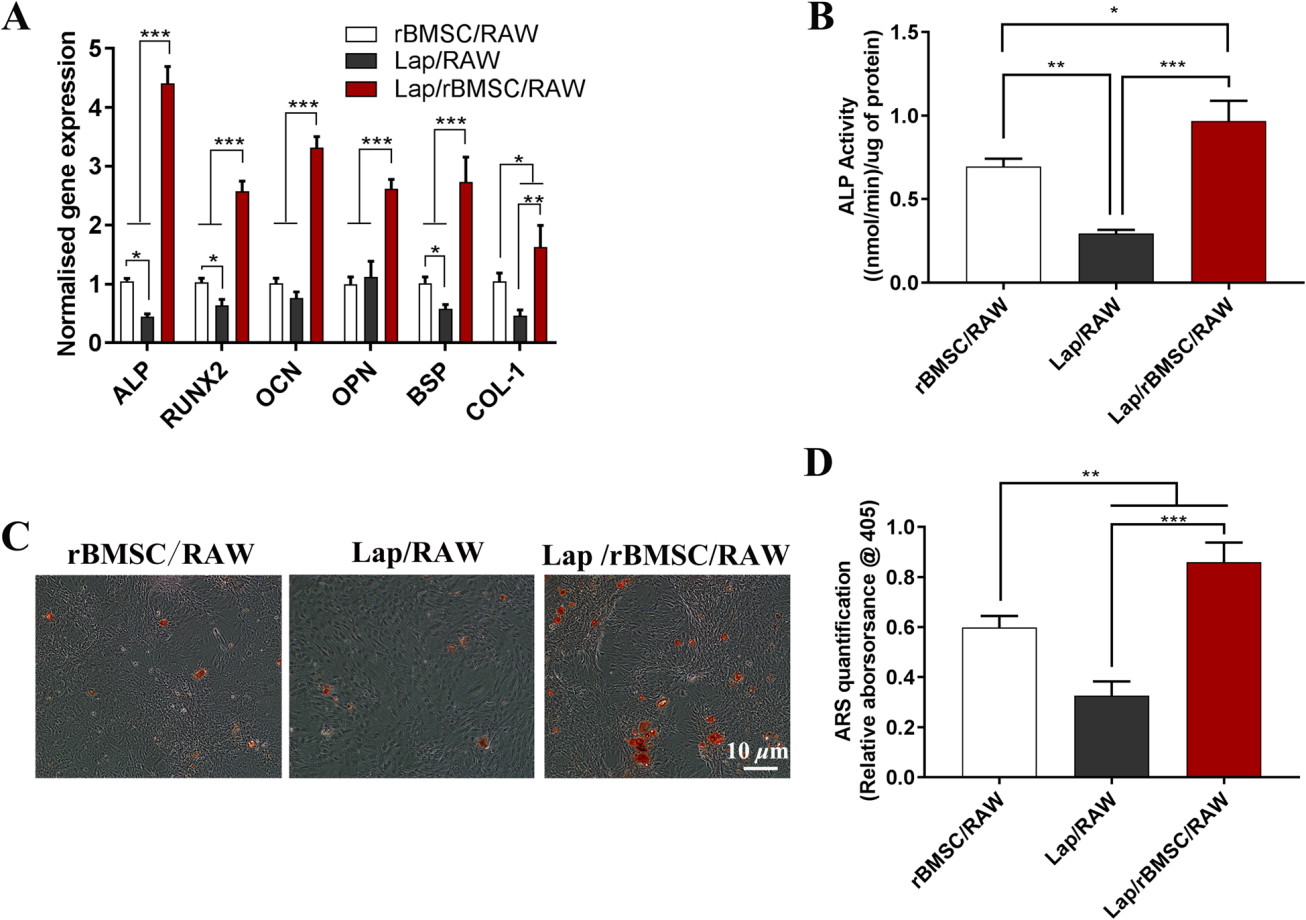
Effect of macrophage-conditioned medium from the rBMSC/RAW, Lap/RAW, and the Lap/rBMSC/RAW on the osteogenic differentiation of rat bone marrow mesenchymal stem cells (rBMSCs). **a** Expression of osteo-related genes; (**b**) alkaline phosphatase (ALP) activity results; and (**c, d**) Alizarin Red S (ARS) staining and quantitative results. The experiment was repeated three times with similar results. *P < 0.05, **P < 0.01, ***P < 0.001. Lap laponite

As shown in Fig. 8a, the mRNA expression of osteo-related genes (*ALP, RUNX-2, OCN,* and* OPN*, as well as *BSP* and *COL-1*) was obviously increased in rBMSCs stimulated by the MΦCM of the Lap/rBMSC/RAW group compared with that stimulated by the MΦCM of the Lap/RAW or rBMSC/RAW groups. The ALP activity, Alizarin Red S staining, and quantitative analysis showed similar trends in that the Lap/rBMSC/RAW group could induce more effective osteogenesis (Fig. 8b–d).

#### Effects of Lap on rBMSC osteogenesis in vivo

**Fig. 9:**
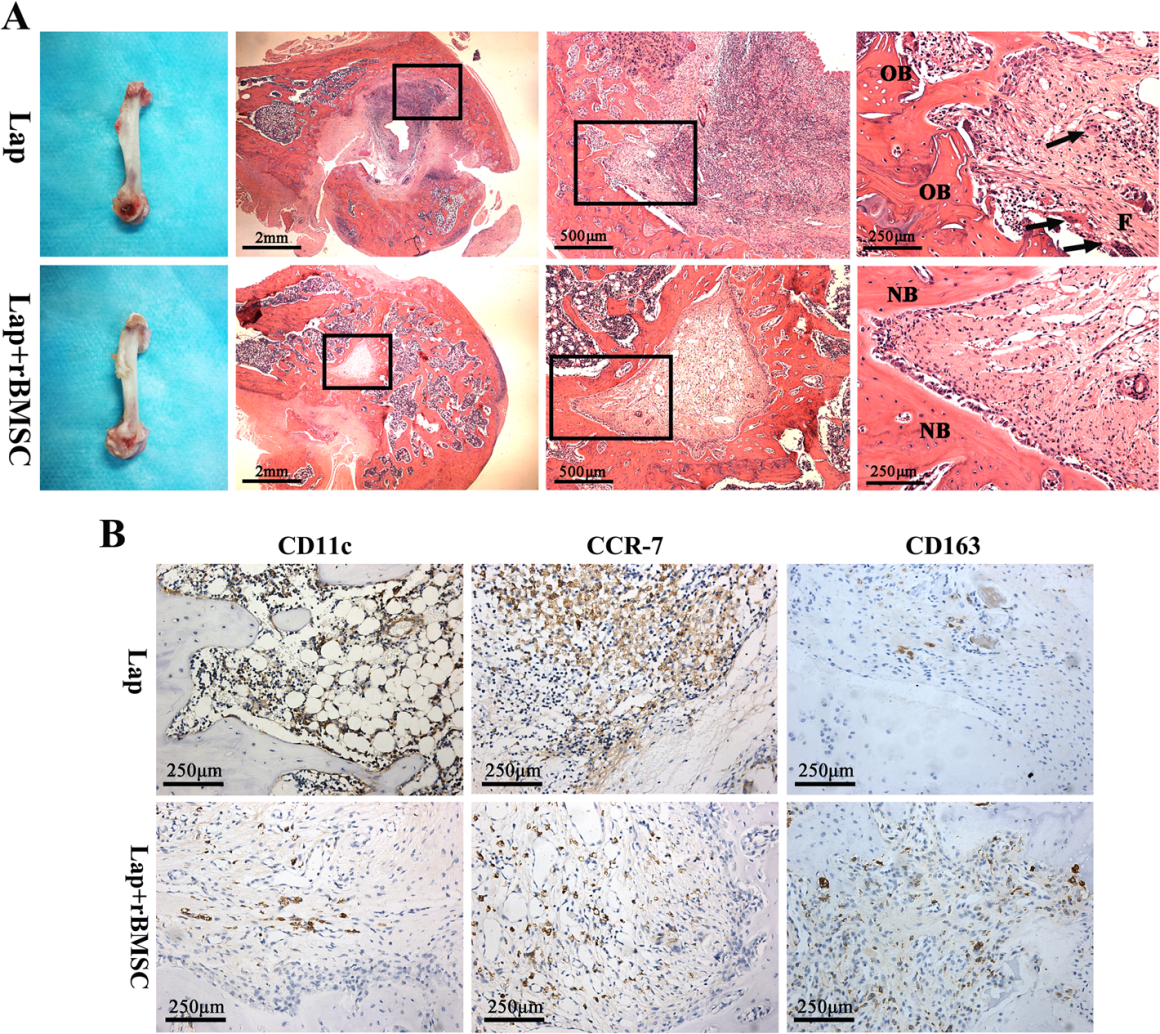
**a** The gross view and H&E staining of the defects filled with laponite (Lap) and Lap + rat bone marrow mesenchymal stem cells (Lap+rBMSCs). **b** Immunohistochemical staining of the surface markers for M1 macrophages (CD11c and CCR-7) and M2 macrophages (CD163). The experiment was repeated three times with similar results. The arrows show multinucleated giant cells. F fibrous tissue, NB newly formed bone, OB old bone

In order to investigate whether the combination of rBMSCs with Lap could improve bone regeneration through altering macrophage polarization, an in vivo study was performed to determine the role of macrophages in bone regeneration. The global view showed almost complete repair of the bone defect in the Lap+rBMSC group, whereas the defect treated with Lap alone showed no evidence of healing (Fig. 9a). H&E staining (Fig. 9a) of the tissue sections from the rat femoral condyle defect model showed obvious new bone formation in Lap+rBMSC group, whereas no obvious new bone formation was detected in the pure Lap group. In addition, fibrous inflammatory tissue and an abundance of multinucleated giant cells could be observed in the bone defect in the Lap group which may be due to the sustained inflammatory stimulus of Lap (Fig. 9a). To confirm the occurrence of macrophage polarization around the Lap and rBMSC/Lap implant sites, immunohistochemical staining of M1 (CCR-7, CD11c) and M2 (CD163) markers was performed to examine the predominant phenotype switch (Fig. 9b). More CCR-7 and CD11c (M1)-positive stained cells were found in the Lap group when compared with the Lap+rBMSC group. Conversely, a significant increase in CD163 (M2)-positive cells was observed in the Lap+rBMSC group than in the Lap group.

## Discussion

Immune reactions following implantation in vivo is vital to the prognosis of biomaterials in BTE and a new in-vitro assessment approach with inclusion of macrophages has gradually become the main evaluating criterion for the osteogenesis of new fabricated materials [2, 8]. Along with advances in the understanding of the divergent effects of distinct subtypes of macrophages (M1 and M2) in tissue regeneration, more and more attention has focused on the strategies for modulating the immune microenvironment around the implants [32]. The immunomodulatory properties of MSCs on macrophages provides a new means for promoting tissue regeneration and it has been suggested that it be incorporated in design of tissue engineering materials [33, 34]. However, studies concerning the immunomodulatory functions of BMSCs in BTE are limited and much remains to be done for the application of BMSCs in a clinical setting.

In the present study, we took advantage of the immunomodulatory capacity of BMSCs to ameliorate the inflammation induced by Lap and to promote its osteogenesis. Our results showed that rBMSCs could reverse the phenotype switch of macrophages induced by Lap, inhibit the proinflammatory cytokines, and promote the production of anti-inflammatory factors as well as osteo-related cytokines. The results of our study demonstrate that BMSCs may represent a new reliable strategy to promote, modulate, or even reverse the “osteoimmunomodulatory” properties of biomaterials in BTE.

### Lap-modulated macrophages

Previous study has shown that after nanoparticles enter the body these foreign materials primarily target the macrophages. Once macrophages engulf the nanoparticles, their polarization and reprogramming can be perturbed, which further influences their immunological properties and cell behavior [35]. This process has been closely related to the characteristics of nanoparticles, such as the diameter, physico-chemistry features, and concentrations [35]. Lap are synthetic disc-shaped crystals with a high aspect ratio and negatively charged surface. When dissolved in water at high concentration (> 100 μg/mL), the overall size of Lap increased significantly. These effects could markedly promote the adherence of Lap to the surface of the cell and intensify the induction of cell stress. In addition, upon formation of aggregates resulting from the interaction of Lap with media proteins that cannot be engulfed by cells, the cytotoxicity of Lap increased and cell metabolic activity was inhibited [24]. It has been reported that silica nanoparticles with high surface reactivity were associated with enhanced interaction with serum proteins and the cell membrane, and caused great oxidative stress and strong proinflammatory effects in macrophages [36]. As demonstrated in our study, as the concentration of Lap increased, and in particular at 100 μg/mL, a significant effect on the morphology and behavior change of the RAW264.7 cells was elicited, as well as the phenotype of macrophages being polarized to the M1 extreme and the inflammatory reaction exacerbated.

### Lap-modulated macrophages reverse the osteogenic effects of Lap

In the present study, when assessed with the traditional “one cell type” approach, the osteogenesis of Lap was significantly enhanced as the concentration increased from 0 μg/mL to 100 μg/mL. However, this trend was disturbed when osteogenesis was assessed with a “multiple cell types” approach. In the new evaluation system, despite being higher than the control group (0 μg/mL Lap), the expression of the osteogenic genes, ALP activity, and mineralized nodule formation of the Lap groups were downregulated as the Lap concentration increased from 12.5 μg/mL to 100 μg/mL. This effect may be mediated in part by the excessive release of proinflammatory cytokines in the 50 and 100 μg/mL groups after macrophage involvement as high levels of proinflammatory cytokines that impede osteogenic differentiation [3]. Previous studies have shown that pre-osteoblast cells differentiated toward a fibroblastic phenotype when cultured with osteogenic medium mixed with the MΦCM from LPS-activated M1 inflammatory macrophages and that this effect may result from the enhanced expression of proinflammatory cytokines (IL-1β, IL-6, TNF-α, IFN-γ) [3, 37]. Additionally, increased levels of M1 macrophages have also been implicated in vivo in animal models of rheumatoid arthritis and other bone diseases where the excessive production of inflammatory factors is thought to be responsible for the failure of MSC-based bone tissue regeneration [11, 38, 39]. Notably, in the present study, the osteogenesis of rBMSCs in the 12.5 μg/mL Lap group was significantly higher than in the other groups. This may be attributed to the following: first, compared with the other groups, flow cytometry implicated less M1 polarization in the 12.5 μg/mL Lap group with a higher expression of anti-inflammatory factors such Arg-1 and IL-10 which are beneficial for osteogenesis [32, 40]. Second, the production of proinflammatory factors such as IL-1β, TGF-β, and IL-6 is relatively low in the 12.5 μg/mL Lap group. Release of these factors has been demonstrated to promote the osteogenic differentiation of MSCs [41–43].

### rBMSCs reverse the polarization of macrophages

BMSCs have been known to be capable of regulating the immune microenvironment by means of interacting with a wide range of immune cells [44]. Recently, studies have shown that the interaction of BMSCs with macrophages was vital in the anti-inflammatory/immunomodulatory effects of MSCs, with the macrophages educated by BMSCs through coculture being identified as a novel type of alternatively activated macrophage (M2) characterized by low expression of *TNF-α* and *IL-12*, but high levels of* IL-10* and *CD206* [45, 46]. In our study, after coculturing RAW264.7 cells with rBMSCs in the presence of Lap, a significant reversal of macrophage phenotype polarization from the proinflammatory M1 to the anti-inflammatory M2 was observed, along with an enhanced expression of anti-inflammatory factors as well as decreased expression of the proinflammatory factors. The crosstalk between MSCs and macrophages is complex, and their communication may develop a feedback loop which not only influences the immune properties of macrophages but also the regenerative potentials of MSCs [32]. Previous research demonstrated that the immunosuppressive capacity of MSCs required stimulation from inflammatory factors, such as IFN-γ and TNF-α [43, 47, 48]. These proinflammatory factors, which have been traditionally recognized as the initiator of macrophage polarization to M1, instead also stimulated the production of immunomodulatory factors such as PEG2 when cocultured with BMSCs. As one of the major factors involved in the immunomodulatory properties of MSCs, PEG2 upregulated the secretion of IL-10 and inhibited the production of cytokines such as TNF-α and IL-12, regulating macrophage polarization and reducing inflammation [49, 50]. In our study, the addition of Lap alone stimulated RAW264.7 cells toward higher production of TNF-α, whereas the additional inclusion of rBMSCs led to the significantly enhanced expression of PEG2 and IL-10 and reduced TNF-α and IFN-γ as detected in the culture medium, accompanied with a switch of macrophage phenotypes from M1 to M2.

### rBMSCs recover the osteogenesis ability of conditioned media of macrophages stimulated by Lap

Several studies have reported that the phenotype of macrophages polarized by MSCs resembles that of alternatively activated macrophages, along with an increased production of IL-10, and reduced production of TNF-α and IL-6 which created an environment promoting bone formation and tissue repair [45, 49, 51]. In our study, we found that, compared with the pure Lap, the immune environment created by Lap after coculture with rBMSCs was more conducive to osteogenesis, with higher expression of osteogenesis-related genes, enhanced ALP activity, and more obvious mineralized nodule formation in vitro. In addition, significant new bone formation rather than inflammatory tissue was observed in vivo in the Lap+rBMSC group. This implied that the introduction of BMSCs recovered the osteogenesis of Lap in a “multiple cells” evaluation approach, and that the inconsistent results of the osteogenic capacity of Lap in “one cell” and “multiple cells” evaluation approaches could be rectified when BMSCs were factored in. Thus, it may be concluded that the use of BMSCs may act as a new strategy in modulating the osteoimmunomodulatory properties of biomaterials.

To ascertain the improved osteogenesis capacity of MΦCM of Lap/rBMSC/RAW, we measured the levels of several factors that may be involved in the process of the coculture system. OSM, one of the IL-6 family cytokines, has been reported to stimulate *STAT3* phosphorylation with enhanced osteogenesis of MSCs by the upregulation of *ALP *and *RUNX2* [31]. Although it remains controversial whether the M1 or M2 phenotype is beneficial for osteogenesis, it has been concluded that activation of the OSM pathway is closely related to the macrophage-modulated osteogenesis [43]. In the present study, the production of OSM in the group of Lap/ rBMSC/RAW264.7 cells was significantly enhanced compared with the other two groups. In addition, the expression of genes (*OSMR, IL-6st, *and *STAT3*) of the OSM signaling pathway in rBMSCs were all enhanced when stimulated by the MΦCM of the rBMSC/Lap/macrophage group compared with that of the Lap/RAW264.7 cells MΦCM group. This demonstrated that activation of the OSM pathway was involved in the enhanced osteogenesis of BMSCs by the factors released in the rBMSC/Lap/macrophage MΦCM. Furthermore, other cytokines such as IL-10 and BMP-2 which are involved in the process of macrophage-conducted osteogenesis were also significantly upregulated in the Lap/rBMSC/RAW group than in the other two groups [31]. These cytokines combined with OSM could lead to improved bone formation. Additionally, an excessive inflammatory response may lead to encapsulation of the biomaterials with inflammatory fibrous tissue which would prevent the integration of bone cells with implanted materials and cause failure of the implantation. In the present study, we assessed the gene expression related to fibrous inflammatory factors (TGF-β1, TGF-β3, and VEGF) [37, 52] and showed significantly greater upregulation in the macrophage-conditioned Lap group than the control group, indicating that the immune microenvironment induced by Lap was able to promote the formation of inflammatory fibrosis, as confirmed by the in vivo results. In contrast, this scenario was reversed when rBMSCs were factored in, such that the gene expression of *TGF-β1* and *TGF-β3* were downregulated.

## Conclusion

In summary, Lap was found to function as a potential bone biomaterial when evaluated with the traditional “one cell” strategy; however, this effect was attenuated when the evaluating system changed to a “multiple cell types” approach to assess osteogenesis with inclusion of macrophages. Instead, the addition of Lap resulted in the M1 polarization of macrophages, and an excessive production of proinflammatory factors that impeded new bone formation. The strategy of incorporating BMSCs to reverse the osteoimmunomodulatory properties of Lap was proven to be feasible, resulting in a less severe immune response and improved bone repair. In the future, additional research should be performed to investigate the role of MSCs and their mechanism in promoting the osteogenesis of biomaterials by regulating the immune response. Concurrently, when evaluating osteogenesis stimulated by bone biomaterials in vitro and in vivo, the assessment of more detailed and comprehensive immune responses is required to investigate the involvement of the interaction between MSCs and other immunocytes such as lymphocytes, dendritic cells, granulocytes, and mast cells.
